# Catalysis over zinc-incorporated berlinite (ZnAlPO_4_) of the methoxycarbonylation of 1,6-hexanediamine with dimethyl carbonate to form dimethylhexane-1,6-dicarbamate

**DOI:** 10.1186/1752-153X-1-27

**Published:** 2007-11-07

**Authors:** Da-Lei Sun, Jian-Ru Deng, Zi-Sheng Chao

**Affiliations:** 1 College of Chemistry and Chemical Engineering, Hunan University, Changsha,410082 People's Republic of China; 2 Key Laboratory of Chemometrics & Chemical Biological Sensing Technologies, Ministry of Education, Hunan University, Changsha, 410082 People's Republic of China

## Abstract

**Background:**

The alkoxycarbonylation of diamines with dialkyl carbonates presents promising route for the synthesis of dicarbamates, one that is potentially 'greener' owing to the lack of a reliance on phosgene. While a few homogeneous catalysts have been reported, no heterogeneous catalyst could be found in the literature for use in the synthesis of dicarbamates from diamines and dialkyl carbonates. Because heterogeneous catalysts are more manageable than homogeneous catalysts as regards separation and recycling, in our study, we hydrothermally synthesized and used pure berlinite (AlPO_4_) and zinc-incorporated berlinite (ZnAlPO_4_) as heterogeneous catalysts in the production of dimethylhexane-1,6-dicarbamate from 1,6-hexanediamine (HDA) and dimethyl carbonate (DMC). The catalysts were characterized by means of XRD, FT-IR and XPS. Various influencing factors, such as the HDA/DMC molar ratio, reaction temperature, reaction time, and ZnAlPO_4_/HDA ratio, were investigated systematically.

**Results:**

The XRD characterization identified a berlinite structure associated with both the AlPO_4 _and ZnAlPO_4 _catalysts. The FT-IR result confirmed the incorporation of zinc into the berlinite framework for ZnAlPO_4_. The XPS measurement revealed that the zinc ions in the ZnAlPO_4 _structure possessed a higher binding energy than those in ZnO, and as a result, a greater electron-attracting ability. It was found that ZnAlPO_4 _catalyzed the formation of dimethylhexane-1,6-dicarbamate from the methoxycarbonylation of HDA with DMC, while no activity was detected on using AlPO_4_. Under optimum reaction conditions (i.e. a DMC/HDA molar ratio of 8:1, reaction temperature of 349 K, reaction time of 8 h, and ZnAlPO_4_/HDA ratio of 5 (mg/mmol)), a yield of up to 92.5% of dimethylhexane-1,6-dicarbamate (with almost 100% conversion of HDA) was obtained. Based on these results, a possible mechanism for the methoxycarbonylation over ZnAlPO_4 _was also proposed.

**Conclusion:**

As a heterogeneous catalyst ZnAlPO_4 _berlinite is highly active and selective for the methoxycarbonylation of HDA with DMC. We propose that dimethylhexane-1,6-dicarbamate is formed *via *a catalytic cycle, which involves activation of the DMC by a key active intermediate species, formed from the coordination of the carbonyl oxygen with Zn(II), as well as a reaction intermediate formed from the nucleophilic attack of the amino group on the carbonyl carbon.

## 1. Background

Organic carbamates are important raw materials and/or intermediates in the production of fine chemicals [1], such as pesticides, herbicides, pharmaceuticals and dyestuffs [2,3], in addition to being employed as amine protecting groups in organic synthesis [1] and linkers in combinatorial synthesis [1,4].

The conventional process for the production of organic carbamates is based mainly on the reaction between alcohols and toxic isocyanates [5]. This approach is not environmentally friendly, because of the need to input high amounts of energy, the use of toxic phosgene as a raw material and also the formation of corrosive hydrochloric acid as a side product [2,6]. Several phosgene-free processes for the production of organic carbamates, such as reductive carbonylation of nitro compounds [7], oxidative carbonylation of amines [8], and alcoholysis of substituted urea [9-11], have been explored. Before these processes acquire a real industrial application, much work has to be carried out in order to reduce the severity of the reaction conditions employed, which presently require high temperatures and pressures, and the use of expensive noble metal catalysts. Recently, an alternative route to organic carbamates *via *the alkoxycarbonylation of amines with alkyl carbonates [12-14] – especially dimethyl carbonate (DMC) – under relatively mild reaction conditions and using cheap catalysts, has been receiving considerable attention. This route appears promising, also given that DMC is presently produced on a large scale by the oxidative carbonylation of methanol [15]. In addition, DMC is easy to handle, cheap, nontoxic and clean, with the methoxycarbonylation reaction yielding methanol as main byproduct. Thus, if the methoxycarbonylation reaction between amines and DMC is coupled with the oxidative carbonylation of alcohols, it is hoped that a green, "zero emission" process for the production of organic carbamates, with could be achieved.

Dialkyl carbamates are of significant importance in industry because their thermolysis results in diisocyanates, which are precursors of polyurethane [16]. A few reports have dealt with the synthesis of dialkyl carbamates from the alkoxycarbonylation reaction between diamines and DMC using homogeneous catalysts [17,18]. However, difficulties associated with homogeneous catalysis systems are well known, notably those relating to the separation and recycling of the catalyst that result in loss of the latter and also product contamination. These issues can be overcome easily if a heterogeneous, rather than homogeneous, catalyst is employed. Regarding the synthesis of dialkyl carbamates from the reaction between diamines and DMC, to the best of our knowledge, no heterogeneous catalyst has been hitherto reported.

Berlinite is the most stable and nonporous phase of all polymorphs of aluminophosphates [19]. Its main potential application can be seen in the field of functional materials, such as high-performance sealants of corrosion- and wear-resistant coatings [20,21], acoustic wave devices, memory glass [22] and piezoelectric materials [23]. While porous aluminophosphates and their transition metal-incorporated derivatives were widely used as catalysts [24,25], a catalytic application of berlinite was never found.

In this study, we address for the first time the preparation of a zinc-incorporated berlinite, namely ZnAlPO_4 _– using 1,6-hexanediamine (HDA) as a structural template – and its application as a heterogeneous catalyst in the production of dimethylhexane-1,6-dicarbamate *via *the methoxycarbonylation between HDA and DMC under mild conditions. Factors influencing the reaction were studied systematically, with a possible reaction mechanism also proposed.

## 2. Experimental

### 2.1. Synthesis of the ZnAlPO_4 _catalyst

Zn(OAc)_2 _(A.R., Shanghai Zhenxin chemical reagent factory), Al(OAc)_3 _(A.R., Beijing chemical reagent company), and H_3_PO_4 _(85%, Tianjin Kermel chemical reagent company) were used as inorganic resource species and HDA (A.R., Shanghai Lingfeng chemicals company) as the structure-directing template.

The ZnAlPO_4 _catalyst was synthesized as follows: Al(OAc)_3 _was first hydrolyzed at room temperature for 2 h and then mixed homogeneously with an aqueous solution of Zn(OAc)_2 _and H_3_PO_4_. The resulting mixture was then aged at room temperature for 2 h, before the addition of HDA at 273 K under vigorous stirring, resulting in a gel with a molar composition of 0.05 Zn:1.78 Al:2.0 P:1.0 HDA:50 H_2_O. The gel was stirred at 273 K for 3 h and then charged into a Teflon-lined autoclave. After hydrothermal crystallization at 453 K for 24 h, the ZnAlPO_4 _solid specimen was recovered by filtration, washed with deionized water until neutrality, and dried. To remove the HDA template, the ZnAlPO_4 _was calcined at 823 K for 8 h.

As a comparison, an AlPO_4 _catalyst was also prepared using the same procedure as that used for ZnAlPO_4_, but in the absence of Zn(OAc)_2_.

### 2.2. Characterization of the AlPO_4 _and ZnAlPO_4 _catalysts

X-ray diffraction (XRD) spectroscopy was performed on a Brucker D8 Advance diffractometer with Cu K*α*1 radiation. Scans were made over a range of 2θ = 6–80°, at a rate of 1°/min.

Fourier transform infrared (FT-IR) spectroscopy was conducted on a Varian 3100 spectrometer, and operated in transmission mode at a resolution of 4 cm^-1^. The catalyst specimen was mixed with KBr in a weight ratio of 1:200, and pressed into pellet to be used in the measurement. The spectrum was recorded as an accumulation of the results of 125 scans, with that of dry KBr subtracted as the background.

X-ray photoelectron spectroscopy (XPS) was carried out on a Phi Quantum 2000 Scanning ESCA Microprobe with Al K*α *radiation. A C_1s _binding energy of 284.6 eV was used as a reference.

### 2.3. Methoxycarbonylation of HDA with DMC

The methoxycarbonylation of HDA with DMC was performed under an N_2 _atmosphere in a 250 mL three-neck flask equipped with a condenser and a magnetic stirrer. In the typical procedure, certain amounts of the reactants, HDA and DMC, and the AlPO_4 _or ZnAlPO_4 _catalyst were added into the three-neck flask at room temperature, before the introduction of a nitrogen flow to drive out the air contained in the flask. The DMC acted as both reactant and solvent. The reaction was carried out at a target temperature, and then, to study the effect of reaction time, the reaction product was sampled periodically. Unless otherwise stated, the reaction can be assumed to have been carried out at 353 K for 8 h, employing 200 mmol of HDA, 1.0 g of ZnAlPO_4 _catalyst and a DMC/HDA molar ratio of 8:1. After the reaction, the catalyst was separated from the reaction products by filtration. The filtrate was first vacuum-distillated to remove the unreacted DMC, and dried at 373 K, resulting in a solid. The solid was then dissolved in methanol and the resulting solution subjected to chromatographic analysis. The qualitative analysis was performed on a Thermo-Finnigan LCQ-Advantage LC/MS^n^. The quantitative analysis was carried out on an Agilent 1100 Series HPLC instrument equipped with a 125 × 4.0 mm Hypersil ODS (C-18) column and an ultraviolet detector. The analysis conditions were flow phase V(methanol):V(water) = 60:40, a flow rate of 1.0 ml/min, column temperature of 298 K, and ultraviolet wavelength of 208 nm. The content of dimethylhexane-1,6-dicarbamate in the reaction product was determined using an external standard method and calculated using the equation W_*sp *_= W_*st*_·A_*sp*_/A_*st *_× 100%, where *sp *and *st *refer respectively to specimen and standard. The conversion of HDA and the yield of dimethylhexane-1,6-dicarbamate were calculated based on the quantity of converted HDA. The selectivities to the components in the reaction product were calculated using the area normalization method.

## 3. Results and discussion

### 3.1. Structural characterization of the catalyst

**Figure 1:**
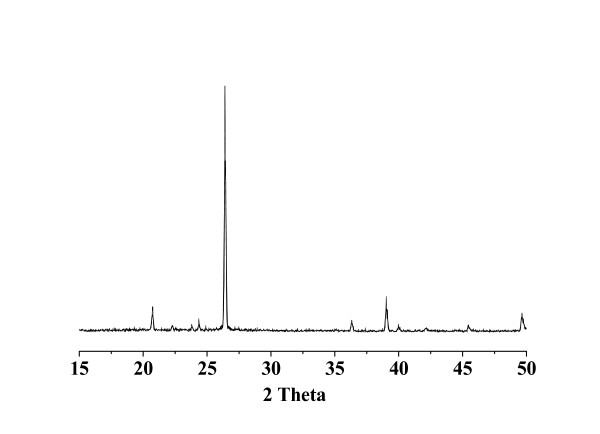
The XRD pattern for ZnAlPO_4_.

The XRD patterns of the ZnAlPO_4 _(Figure 1) and AlPO_4 _(same as Figure 1, but not given) are totally consistent with those of standard berlinite (JCPDS No. 76-227). No other crystalline and amorphous phases were detected. It can be stated that both the ZnAlPO_4 _(Figure 1) and AlPO_4 _catalysts prepared in this work possess a pure berlinite structure.

**Figure 2:**
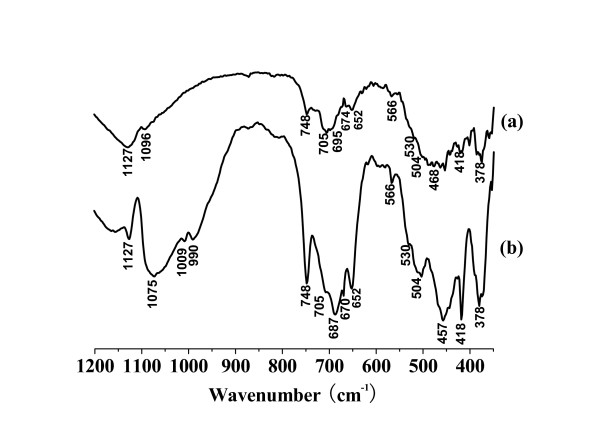
The FT-IR spectrum of (a) AlPO_4 _and (b) ZnAlPO_4_.

Figure 2 shows the FT-IR spectra of the AlPO_4 _and ZnAlPO_4 _catalysts. The spectrum of the AlPO_4 _(Figure 2a) exhibited the characteristic vibration absorptions of a berlinite structure [19,26-29], i.e. the bands associated with vibrations of the [PO_4_]^3- ^unit (1127, 1096, 530, 504, 468, 418, and 378 cm^-1^), those of the pseudolattice Al vibrations (705, 687, 670, 652, and 566 cm^-1^), and a band (748 cm^-1^) ascribed to an overtone of that at 378 cm^-1 ^(or, a result of sub-lattice aluminium defects in berlinite). The spectrum of ZnAlPO_4 _(Figure 2b) also presented the above bands, some of which were shifted towards lower wavenumbers probably due to the incorporation of Zn into the berlinite framework. In addition, two additional bands at 1009 and 990 cm^-1 ^were also detected in the ZnAlPO_4_spectrum compared to that for AlPO_4_. Thus, the bands at 1009 and 990 cm^-1 ^should be caused by the incorporation of Zn into the berlinite framework and assigned to the vibrations of Zn-O-P [27,28]. Infrared bands characteristic of ZnO are only observed below 600 cm^-1 ^and a broad band at 400–550 cm^-1^had been reported by Oliver [28]. For ZnAlPO_4_, the sharp bands – rather than broad bands – attributed to [PO_4_]^3- ^vibrations were observed in the range of 300–550 cm^-1^, providing further evidence for the incorporation of Zn into the berlinite framework.

The XPS measurement shows that the atomic composition of the surface of ZnAlPO_4 _is composed of Zn:Al:P:O = 1.0 2.9:3.2:15.6. The binding energy of Zn_2p3/2 _for ZnAlPO_4 _was found to be 1022.3 eV, slightly higher than that for ZnO (1021.8 ± 0.3 eV [30]). This indicates that Zn(II) ions are incorporated into the berlinite framework, and possess a higher tendency to draw electrons as compared to those in ZnO.

### 3.2. The reaction between HDA and DMC

AlPO_4 _and ZnAlPO_4 _had been employed as the catalyst for the reaction between HDA and DMC. We found that AlPO_4 _presented no catalytic activity, while ZnAlPO_4 _did have a catalytic effecr. It was found that, in addition to the main product, dimethylhexane-1,6-dicarbamate (dicarbamate), two by-products, methyl-6-amino-hexane-carbamate (monocarbamate) and methyl-6-methylamino-hexane-carbamate (N-methylated-carbamate) were also formed using the ZnAlPO_4 _catalyst and reaction conditions tested in this work. In the following sections, the factors influencing the reaction between HDA and DMC, including DMC/HDA molar ratio, reaction temperature, reaction time and ZnAlPO_4_/HDA ratio (mg/mmol), are examined.

#### 3.2.1. Effect of DMC/HDA molar ratio

**Figure 3:**
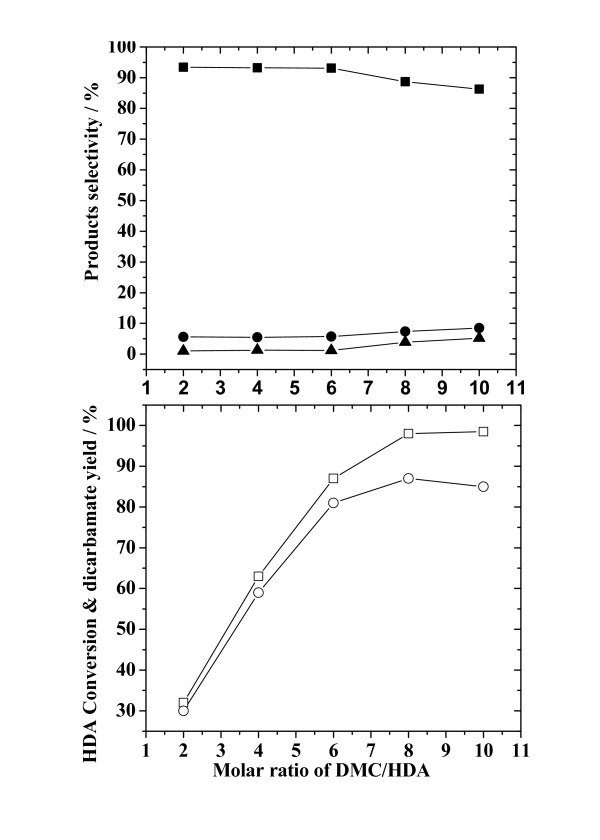
**Effect of the DMC/HDA molar ratio on HDA conversion, yield of dicarbamate and selectivity for reaction products**. Reaction conditions: HDA, 200 mmol; ZnAlPO_4_, 1.0 g; timeframe, 8 h; temperature, 353 K. (Legend: (□) HDA conversion; (○) yield of dicarbamate; (■), (●) and (▲) denote selectivity for dicarbamate, monocarbamate and N-methylated-carbamate, respectively.)

Figure 3 presents the effect of the DMC/HDA molar ratio on HDA conversion and yield of dicarbamate, in addition to selectivities for both the main- and by-products. On increasing the DMC/HDA molar ratio from 2:1 to 8:1, the conversion of HDA and the yield of dicarbamate increased rapidly. Subsequently the HDA conversion increased at a smaller rate and reached a value of as high as 99% when DMC/HDA = 10:1. The yield of dicarbamate increased, followed by a decrease, with the DMC/HDA increasing from 8:1 to 10:1. The maximum yield of dicarbamate was 87%, obtained at a DMC/HDA molar ratio of 8:1. The selectivities for dicarbamate, monocarbamate and N-methylated-carbamate were almost unchanged for HDA/DMC molar ratios over the range of 2:1 to 6:1, while that for dicarbamate decreased. Selectivities for monocarbamate and N-methylated-carbamate increased when DMC/HDA > 6:1. For all the HDA/DMC ratios tested, the selectivity for the main product (dicarbamate) was much larger than for the by-products (monocarbamate and N-methylated-carbamate). It appears that a higher DMC/HDA ratio was favorable for the conversion of HDA and the formation of dicarbamate, but too high a ratio led to a decrease in the yield of dicarbamate. Thus, the optimum DMC/HDA ratio appears to be in the range of 6:1 to 8:1.

#### 3.2.2. Effect of reaction temperature

**Figure 4:**
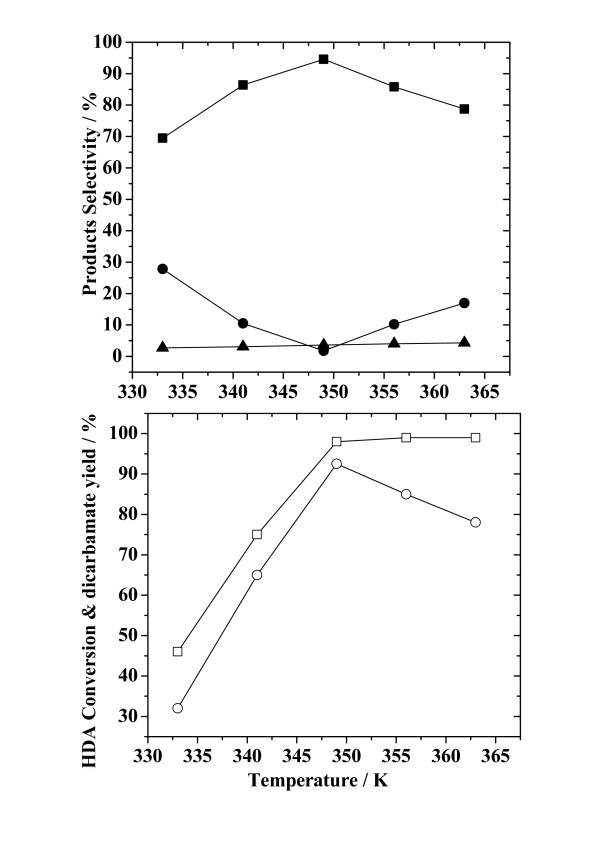
**Effect of the reaction temperature on HDA conversion, yield of dicarbamate and selectivity for reaction products**. Reaction conditions: HDA, 200 mmol; ZnAlPO_4_, 1.0 g; DMC/HDA, 8; time, 8 h. (Legend: (□) HDA conversion; (○) yield of dicarbamate; (■), (●) and (▲), selectivity for dicarbamate, monocarbamate and N-methylated-carbamate, respectively.)

Figure 4 presents the effect of reaction temperature on HDA conversion, yield of dicarbamate and selectivities for both the main- and by-products. On increasing reaction temperature, the conversion of HDA increased rapidly over the temperature range 333 to 349 K, and only slightly at temperatures higher than 349 K, approaching its maximum of 98%. The yield of dicarbamate increased with on moving from 333 to 349 K, attaining a maximum of 92.5% at 349 K, before decreasing at higher temperatures. The selectivity for dicarbamate first increased and then decreased, on increasing the reaction temperature. The maximum selectivity for dicarbamate was achieved at 349 K. The selectivity for monocarbamate presented an inverse trend with reaction temperature, compared with that for dicarbamate, and achieved a minimum value at 349 K. The selectivity for N-methylated-carbamate was almost unchanged with reaction temperature. It appears that the decrease in selectivity for dicarbamate at temperatures higher than 349 K was due to the increase in that for monocarbamate. For all the reaction temperature points tested, the selectivity for various products was: dicarbamate >> monocarbamate > N-methylated-carbamate. The above results indicate that the elevation of reaction temperature promoted the conversion of HDA. Although a higher conversion of HDA could be attained at high temperature, too high a temperature reduced the yield of dicarbamate – possibly due to the partial decomposition of dicarbamate into monocarbamate. Thus, the optimum reaction temperature for the production of dicarbamate from the methoxycarbonylation of HDA with DMC is around 349 K.

#### 3.2.3. Effect of reaction time

**Figure 5:**
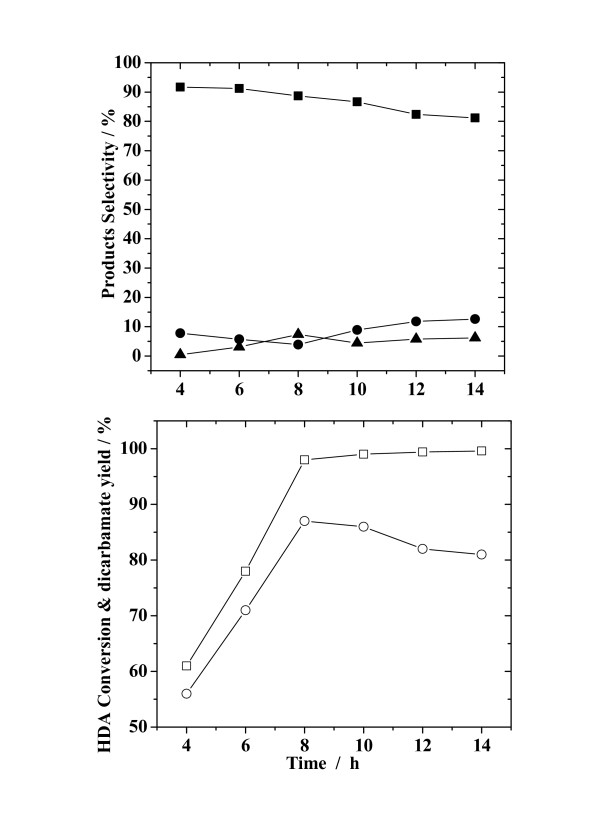
**Effect of reaction time on HDA conversion, yield of dicarbamate and selectivity for reaction products**. Reaction conditions: HDA, 200 mmol; ZnAlPO_4_, 1.0 g; DMC/HDA, 8; temperature, 353 K. (Legend: (□) HDA conversion; (○) yield of dicarbamate; (■), (●) and (▲), selectivity for selectivity for dicarbamate, monocarbamate and N-methylated-carbamate, respectively.)

Figure 5 outlines the effect of reaction time on HDA conversion, yield of dicarbamate and selectivities for both the main- and by-products. With increasing reaction time, the HDA conversion increased quickly within 8 h and only slightly over longer reaction times, reaching a value of nearly 100%. The yield of dicarbamate increased, followed by a decrease, with a maximum value of 87% being achieved at a reaction time of 8 h. For all the reaction timeframes tested, the selectivity for dicarbamate was much higher than those for monocarbamate and N-methylated-carbamate. On prolonging the reaction timeframe, the selectivity for dicarbamate decreased gradually. The selectivity for monocarbamate presented a minimum and that for N-methylated-carbamate a maximum at a reaction time of 8 h. Thus the decrease in the yield of dicarbamate at a longer reaction time may be explained by the partial decomposition of dicarbamate to monocarbamate. It appears that too long a reaction time did not promote greater formation of dicarbamate – but rather resulted in a reduced yield. The optimum reaction time is suggested as being 8 h.

#### 3.2.4. Effect of the ZnAlPO_4_/HDA ratio (mg/mmol)

**Figure 6:**
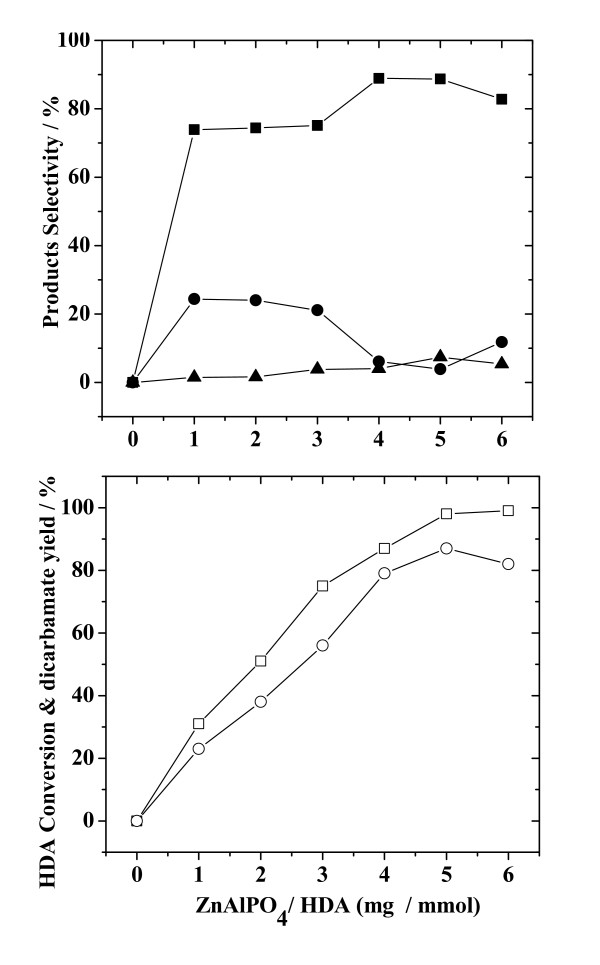
**Effect of the ZnAlPO_4_/HDA ratio (mg/mmol) on HDA conversion, yield of dicarbamate and selectivity for reaction products**. Reaction conditions: HDA, 200 mmol; DMC/HDA, 8; time, 8 h; temperature, 353 K. (Legend: (□) HDA conversion; (○) yield of dicarbamate; (■), (●) and (▲), selectivity for dicarbamate, monocarbamate and N-methylated-carbamate, respectively.)

Figure 6 presents the effect of ZnAlPO_4_/HDA ratio (mg/mmol) on HDA conversion, yield of dicarbamate and selectivities for both the main- and by-products. In the absence of the ZnAlPO_4 _catalyst, no reaction was found to occur between HDA and DMC. When the ZnAlPO_4 _catalyst was employed at a ratio of ZnAlPO_4_/HDA = 1:1 (mg/mmol), dicarbamate was obtained in a 23% yield with an HDA conversion of 31%. The reaction product mixture was identified as consisting of 73.9% dicarbamate, 24.4% monocarbamate and 1.7% N-methylated-carbamate. On increasing the ZnAlPO_4_/HDA ratio, HDA conversion increased, reaching a value of nearly 100%, whilst the yield of dicarbamate increased, followed by a decrease, with a maximum of 87% achieved at the ratio of 5:1. With the ZnAlPO_4_/HDA ratio increasing from 1:1 to 3:1, selectivity for dicarbamate increased slowly while that for monocarbamate decreased. At a ZnAlPO_4_/HDA ratio higher than 3:1, the selectivity for dicarbamate increased rapidly, reaching a maximum of 88.9% at a ratio of 5:1, before decreasing. The selectivity for N-methylated-carbamate was similar but the selectivity for monocarbamate showed an inverse trend, like that observed for dicarbamate. A maximum of 7.4% selectivity for N-methylated-carbamate and a minimum of 3.9% for monocarbamate were obtained at the ZnAlPO_4_/HDA ratio of 5. Thus, a ZnAlPO_4_/HDA ratio of 5 appears to be most favorable for the production of dicarbamate from the methoxycarbonylation of HDA with DMC.

### 3.3. Mechanistic consideration to the methoxycarbonylation of HDA with DMC over the ZnAlPO_4 _catalyst

Hitherto only the use of homogeneous catalysts has been reported in the methoxycarbonylation of diamines with dialkyl carbonates. In this study we employed zinc-incorporated berlinite (ZnAlPO_4_) as a heterogeneous catalyst for the production dimethylhexane-1,6-dicarbamate from the methoxycarbonylation of HDA with DMC. The structure of berlinite is known to be derived from that of quartz, in that Si(IV) is replaced alternately with P(V) and Al(III), with a neutral framework. When Zn(II) ions are incorporated into the framework of berlinite, P(V) and/or Al(III) are replaced, resulting in oxygen vacancies in close vicinity to Zn(II). This enables zinc ions in ZnAlPO_4 _to acquire a higher electron attracting ability than those in ZnO, as evidenced by the XPS results.

The alkoxycarbonylation of amines with dialkyl carbonate results from the nucleophilic attack of the amine on the carbonyl carbon of dialkyl carbonate. While an amine of significantly higher basicity than the leaving group (alkoxyl) is required [6,31-33], the alkoxycarbonylation reaction depends largely upon the reactivity of dialkyl carbonate. Factors that increase the electrophilicity of the carbonyl carbon may therefore promote the reaction, i.e. an electron-withdrawing effect exerted on the carbonyl group facilitates its reaction with nucleophilic amine [6,14,31,34]. Baba [6] studied the methoxycarbonylation of diamines with DMC using Zn(OAc)_2_·2H_2_O as a catalyst. It was identified that the transformation from the bidentate to the monodentate coordination mode of CH_3_COO^- ^anions to zinc ionshelped activate the carbonyl groups of DMC, owing to the coordinated DMC behaving as an active intermediate species, able to react with amines. For the methoxycarbonylation of HDA with DMC over ZnAlPO_4_, an active intermediate species resulting by the coordination of the carbonyl oxygen of DMC to Zn(II) is believed to form. This deduction is based on the following considerations: 1) It was found in this work that the methoxycarbonylation of HDA with DMC occurred only in the presence of ZnAlPO_4_, and not in the presence of the AlPO_4_, revealing that that either HDA or DMC had been activated in some manner over the catalyst; 2) Although as a potential chelating N-donor species [13,35], the aliphatic diamine, may coordinate Zn(II), the basicity of a HDA coordinated to Zn(II) would be expected to be no more than that of free HDA. Thus, the possibility of HDA coordinating Zn(II) and acting as an active species can be excluded. In fact, transition metal ions often possess high oxophilicity in the presence of both a N-donor and O-donor species [35]; 3) The unusually high ability of Zn(II) in ZnAlPO_4 _to attract electrons facilitates the activation of DMC by partially transferring an electron from the DMC carboxyl oxygen to Zn(II).

**Scheme 1:**
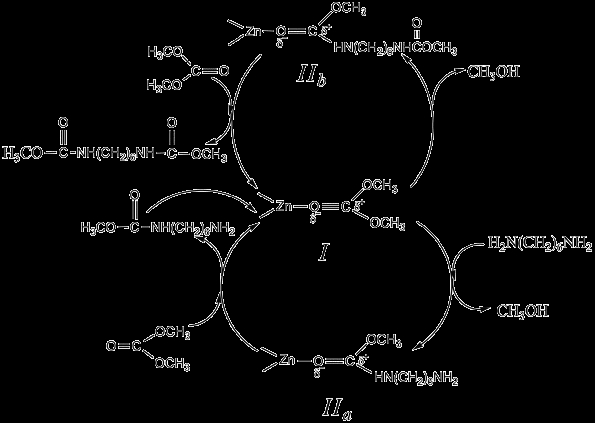
A proposed mechanism for the methoxycarbonylation of 1,6-hexanediamine with DMC over ZnAlPO_4_.

Although mechanistic studies on the methoxycarbonylation of HDA with DMC in the presence of a ZnAlPO_4 _catalyst are still in progress, it can be surmised that the reaction pathway may involve a catalytic cycle that involves a number of steps (Scheme 1). At first, DMC is activated because of its coordination to Zn(II), forming the species *I *as an active intermediate. Then, the carbonyl carbon in is attacked by the nucleophilic HDA, forming a reaction intermediate species *II*_*a *_and methanol as a by-product. The interaction of the zinc ion in species *II*_*a *_with another molecule of DMC results in the formation of monocarbamate and the recovery of the active intermediate species *I*. Similarly, the dicarbamate product may be result from the interaction between DMC and species *II*_*b*_, which is formed by a nucleophilic attack of monocarbamate on the active intermediate species *I*. It must be noted that DMC, which has an ambident electrophilic nature, may react with a nucleophile not only at the carbonyl group to form carbamate, but also at the methyl moiety, forming the N-methylated product [35,36]. The carbonyl carbon, being a better electrophile, is expected to be more reactive with the amine than the methoxyl carbon For this reason, only a very limited amount of N-methylated product was formed during the reaction between HDA and DMC over the ZnAlPO_4 _catalyst.

## 4. Conclusion

The ZnAlPO_4 _material prepared in this work was identified as having a berlinite structure. The FT-IR characterization of the material indicated that zinc had been incorporated into the aluminophosphate framework. Examination by XPS indicated that the zinc ions in ZnAlPO_4 _had a higher binding energy than those in ZnO, suggesting a larger electron withdrawing tendency in the former. As a heterogeneous catalyst, ZnAlPO_4 _exhibits very high activity and selectivity under mild reaction conditions for the methoxycarbonylation of HDA with DMC to form dimethylhexane-1,6-dicarbamate. A yield of up to 92.5% of dimethylhexane-1,6-dicarbamate with nearly 100% conversion of HDA was obtained at 349 K, over 8 h, with DMC/HDA = 8 and ZnAlPO_4_/HDA = 5 (mg/mmol).

The mechanism for the methoxycarbonylation may have resulted from a catalytic cycle involving a key active intermediate species – formed from the coordination of the carbonyl oxygen with Zn(II) – that activates the DMC, in addition to a reaction intermediate formed from the nucleophilic attack of the amino group on the carbonyl carbon.

## Authors' contributions

This study was conceived as a result of discussion between Prof. Z.S. Chao, Prof. J. R. Deng and Dr. D.L. Su. The synthesis and characterization of the ZnAlPO_4 _catalyst and its catalytic performance evaluation were carried out by Dr. D.L. Sun. The spectroscopic analysis was performed by Prof. Z. S, who also proposed the reaction mechanism for the methoxycarbonylation of 1,6-hexanediamine with DMC. The manuscript was first composed by Dr. D.L. Sun and revised largely by Prof. Z.S. Chao. All authors read and approved the final manuscript.
